# Ultramicronized Palmitoylethanolamide in the Management of Sepsis-Induced Coagulopathy and Disseminated Intravascular Coagulation

**DOI:** 10.3390/ijms222111388

**Published:** 2021-10-21

**Authors:** Ramona D’Amico, Francesco Monaco, Rosalba Siracusa, Marika Cordaro, Roberta Fusco, Alessio Filippo Peritore, Enrico Gugliandolo, Rosalia Crupi, Salvatore Cuzzocrea, Rosanna Di Paola, Daniela Impellizzeri, Tiziana Genovese

**Affiliations:** 1 Department of Chemical, Biological, Pharmaceutical, and Environmental Science, University of Messina, 98166 Messina, Italy; rdamico@unime.it (R.D.); rsiracusa@unime.it (R.S.); rfusco@unime.it (R.F.); aperitore@unime.it (A.F.P.); dimpellizzeri@unime.it (D.I.); tgenovese@unime.it (T.G.); 2 Department of Biomedical and Dental Sciences and Morphofunctional Imaging, University of Messina, 98166 Messina, Italy; francesco.monaco@unime.it (F.M.); cordarom@unime.it (M.C.); 3 Department of Veterinary Science, University of Messina, 98166 Messina, Italy; egugliandolo@unime.it (E.G.); rcrupi@unime.it (R.C.)

**Keywords:** disseminated intravascular coagulation, sepsis, coagulation, inflammation, ultramicronized palmitoylethanolamide

## Abstract

Disseminated intravascular coagulation (DIC) is a severe condition characterized by the systemic formation of microthrombi complicated with bleeding tendency and organ dysfunction. In the last years, it represents one of the most frequent consequences of coronavirus disease 2019 (COVID-19). The pathogenesis of DIC is complex, with cross-talk between the coagulant and inflammatory pathways. The objective of this study is to investigate the anti-inflammatory action of ultramicronized palmitoylethanolamide (um-PEA) in a lipopolysaccharide (LPS)-induced DIC model in rats. Experimental DIC was induced by continual infusion of LPS (30 mg/kg) for 4 h through the tail vein. Um-PEA (30 mg/kg) was given orally 30 min before and 1 h after the start of intravenous infusion of LPS. Results showed that um-PEA reduced alteration of coagulation markers, as well as proinflammatory cytokine release in plasma and lung samples, induced by LPS infusion. Furthermore, um-PEA also has the effect of preventing the formation of fibrin deposition and lung damage. Moreover, um-PEA was able to reduce the number of mast cells (MCs) and the release of its serine proteases, which are also necessary for SARS-CoV-2 infection. These results suggest that um-PEA could be considered as a potential therapeutic approach in the management of DIC and in clinical implications associated to coagulopathy and lung dysfunction, such as COVID-19.

## 1. Introduction

Disseminated intravascular coagulation (DIC) is a clinical syndrome with high mortality caused by activation of systemic intravascular coagulation [1]. It usually develops as a critical complication in patients with life-threatening conditions, such as severe infection, severe sepsis, malignancies, severe trauma, placental abruption, and obstetric calamities [2]. In particular, in the last period, DIC has been of particular interest as it represents one of the most frequent consequences of coronavirus disease 2019 (COVID-19) [3]. In March 2020, the WHO declared COVID-19 as a pandemic worldwide that represents a serious public health risk [4]. The high susceptibility of the population is responsible for the raised incidence of the distribution worldwide so that COVID-19 has also become a serious public health problem. The pathological mechanism underlying DIC includes the diffusion of microvascular thrombosis that prevents normal blood supply to organs and causes cell edema and multiple organ failure (MOF) [5,6]. Lung injury is one of the most known complications of DIC [7,8]; indeed, despite substantial research into new therapeutic strategies, the morbidity and mortality rates associated with lung injury induced by DIC remain high [9]. The pathogenesis of DIC is complex, with interplay between the coagulant, inflammatory, and immune pathways [8]. In the last decades, intravenous infusion of lipopolysaccharide (LPS) has gained wide acceptance as an experimental model of DIC [10]. LPS is the main component of endotoxin, released from lysates of Gram-negative bacteria [11]. LPS activates coagulation and subsequent intravascular coagulation, altering the balance between the coagulation system and fibrinolytic system. Moreover, LPS induces monocytes and endothelial cells to release several inflammatory and chemotactic cytokines, which, in turn, triggers a systemic inflammatory response [12,13]. This cross-talk among coagulation and inflammation leads to the microthrombus formation that contributes to the development of organ dysfunction. In particular, the recent outbreak of DIC emphasizes the lethal outcomes associated with lung injury, characterized by diffuse alveolar damage, pulmonary microvascular endothelial cell damage, and even respiratory failure [7].

Currently, heparin is used as therapy (or prophylaxis) in patients with DIC, but high-dose heparin increases the risk of bleeding [14]. Other anticoagulants used in the treatment of DIC are antithrombin, recombinant thrombomodulin, and plasma-derived activated protein C. However, large-scale randomized controlled trials are yet to be performed with important consideration of the timing, dosage, and duration of treatment [15,16,17]. Therefore, a targeted therapeutic approach to reduce inflammation and clotting activation could improve DIC and its comorbidities.

The aim of our study is to investigate the anti-inflammatory properties of palmitoylethanolamide (PEA), an endogenous lipid belonging to autocoid local injury antagonism amides (ALIAmides) family [18], in an experimental model of DIC. Given the lipidic nature of PEA, it may be expected to have limitations in terms of solubility and bioavailability. The micronization and ultramicronization method is applied for bypassing this problem, reducing particle size and, consequently, increasing the bioavailability and solubility, so increasing the dissolution rate [19]. Recent studies have proposed ultramicronization (um)-PEA as a potential adjunct in therapy for COVID-19 patients, thanks to its ability to modulate inflammation and the synthesis of proinflammatory enzymes [20]. This suggests that um-PEA could be included into the COVID-19 multidrug regimen, preventing an increase in immunosuppressant dosage by planning a synergistic therapy between um-PEA and the latter [21].

Therefore, in the current study, we assessed the anti-inflammatory action of um-PEA in the DIC rats resulting from intravascular infused with LPS to understand the pathogenesis of DIC and the consequent lung dysfunction.

## 2. Results

### 2.1. Effect of um-PEA on Blood Coagulation Parameters

**Figure 1:**
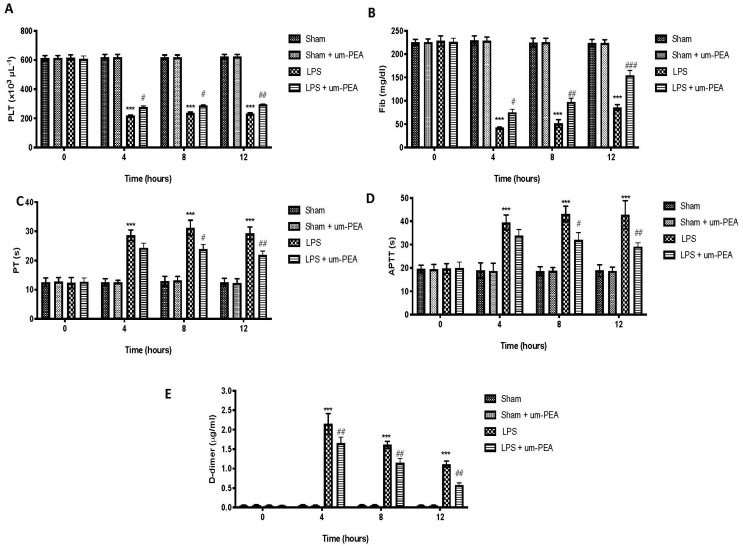
Blood coagulation parameters: platelet (PLT) counts (**A**); fibrinogen (Fib) levels (**B**); prothrombin time (PT) (**C**); activated partial thromboplastin time (APTT) (**D**); D-dimer levels (**E**). *** *p* < 0.001 vs. sham; # *p* < 0.05 vs. LPS; ## *p* < 0.01 vs. LPS; ### *p* < 0.001 vs. LPS.

To investigate the effects of um-PEA on blood coagulation function and fibrinolytic system of rats, we detected the changes in plasma markers for DIC after oral administrations of um-PEA 30 min before and 1 h after the start of infusion of LPS. Plasma platelet (PLT) counts (Figure 1A) and fibrinogen (Fib) concentrations (Figure 1B) in the rats were significantly decreased at 4 h, 8 h, and 12 h after LPS infusion, compared to sham group. In contrast, a simultaneous significant increase in prothrombin time (PT) (Figure 1C), activated partial thromboplastin time (APTT) (Figure 1D), and D-dimer (Figure 1E) levels after LPS administration for 4 h, 8 h, and 12 h was observed. Data showed that um-PEA at the dose of 30 mg/kg significantly increased PLT counts and Fib concentrations, compared to the LPS group (Figure 1A,B, respectively). On the other side, PT and APTT levels were notably reduced in the um-PEA group, compared to LPS animals (Figure 1C,D, respectively). Moreover, plasma D-dimer levels were significantly decreased in the um-PEA group, compared to LPS rats (Figure 1E). These results indicated that um-PEA alleviated systemic intravascular coagulation. No significant changes were found between sham groups.

### 2.2. Effects of um-PEA on Proinflammatory Cytokine Levels

**Figure 2:**
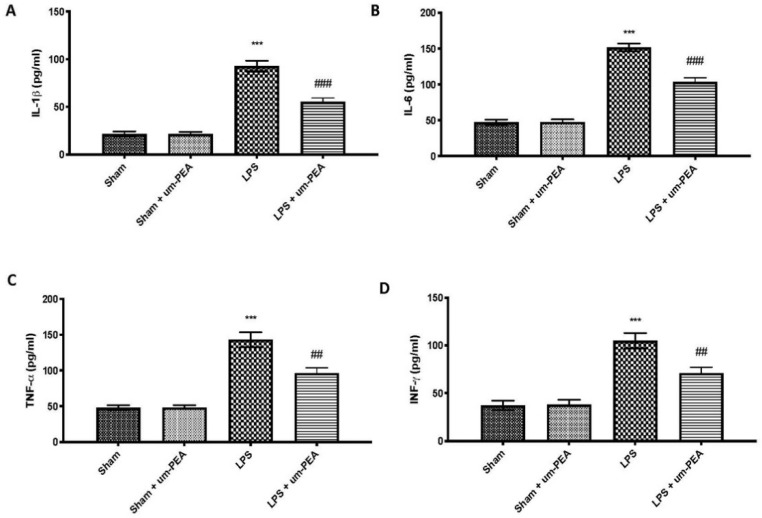
Cytokine levels in plasma: IL-1β (**A**); IL-6 (**B**); TNF-α (**C**); IFN-γ (**D**). *** *p* < 0.001 vs. sham; ## *p* < 0.01 vs. LPS; ### *p* < 0.001 vs. LPS.

To explore the effect of um-PEA on inflammation on DIC, we measured the levels of proinflammatory cytokines in plasma. We found that plasma levels of IL-1β (Figure 2A), IL-6 (Figure 2B), TNF-α (Figure 2C), and IFN-γ (Figure 2D) were markedly increased in the LPS group compared to the control group. In contrast, um-PEA was able to decrease plasma levels of these proinflammatory cytokines.

**Figure 3:**
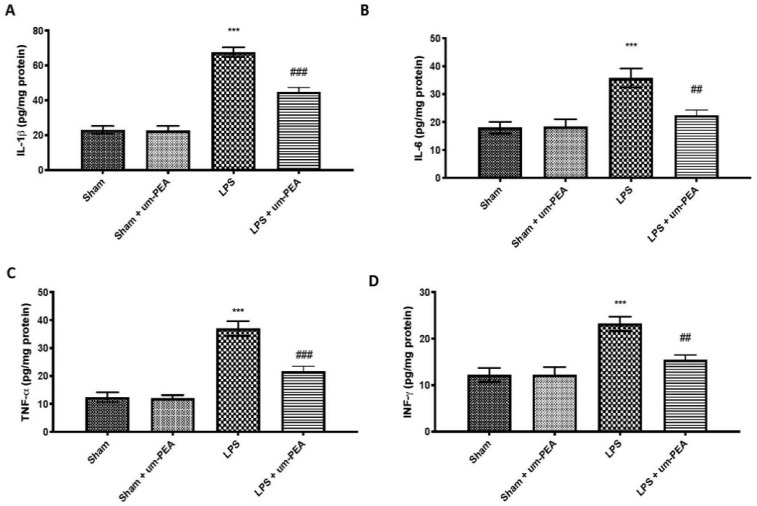
Cytokine levels in lung samples: IL-1β (**A**); IL-6 (**B**); TNF-α (**C**); IFN-γ (**D**). *** *p* < 0.001 vs. sham; ## *p* < 0.01 vs. LPS; ### *p* < 0.001 vs. LPS.

Similar results were observed evaluating the expression of IL-1β (Figure 3A), IL-6 (Figure 3B), TNF-α (Figure 3C), and IFN-γ (Figure 3D) in homogenates of lung samples, further confirming the anti-inflammatory action of um-PEA. No significant changes were found in plasma and lung samples between sham groups.

### 2.3. Effects of um-PEA on Inflammatory Pathway

**Figure 4:**
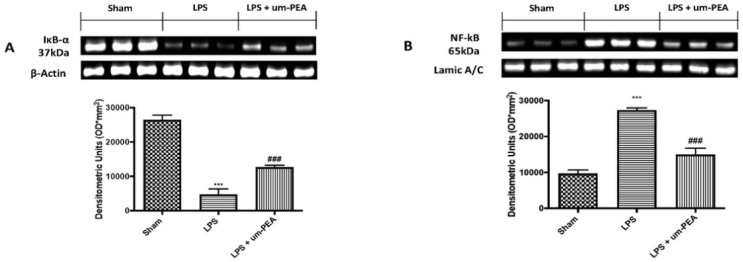
Western blot and, respectively, quantification of IκB-α (**A**) and NF-κB (**B**) in lung tissues. *** *p* < 0.001 vs. sham; ### *p* < 0.001 vs. LPS.

To examine how um-PEA could attenuate the overproduction of proinflammatory mediators, we investigated the NF-kB pathway by Western blot analysis. Since no significant changes were found between the sham groups for coagulation markers and cytokine levels, we present data of the sham + saline group. Our results showed a basal expression of IκB-α in sham rats, while LPS infusion significantly decreased IκB-α expression in lung samples (Figure 4A). At the same time, NF-κB levels were increased significantly in samples from LPS-treated rats compared to the control group (Figure 4B). Um-PEA treatment reduced IKB-α degradation (Figure 4A) and, consequently, nuclear translocation of NF-kB (Figure 4B) induced by DIC.

### 2.4. Effect of um-PEA on the Histological Changes

**Figure 5:**
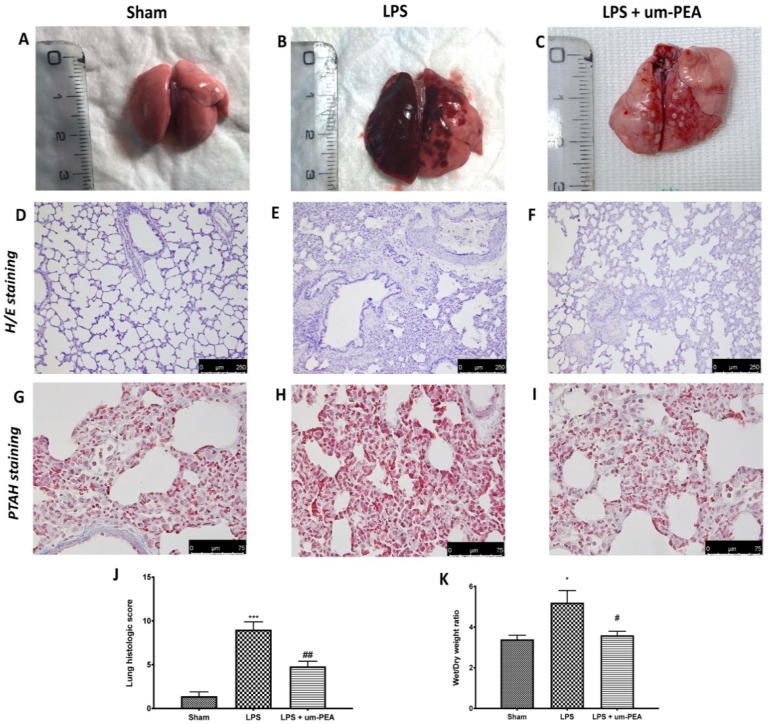
Macroscopic observation: sham (**A**), LPS (**B**), LPS + um-PEA (**C**). H/E staining: sham (**D**), LPS (**E**), LPS + um-PEA (**F**). PTAH staining: sham (**G**), LPS (**H**), LPS + um-PEA (**I**). Histological score (**J**) and wet/dry weight (**K**). A 10× magnification is shown (250-µm scale bar) for H/E staining; a 40× magnification is shown (75-µm scale bar) for PTAH staining. * *p* < 0.05 vs. sham; *** *p* < 0.001 vs. sham; # *p* < 0.05 vs. LPS; ## *p* < 0.01 vs. LPS.

Given that DIC may result in lung dysfunction, we assessed histological injury by macroscopic observation, H/E, and PTAH staining. In the lungs from the control group, no macroscopic change was observed (Figure 5A). After intravenous infusion of LPS, severe hemorrhages were visible on the surface of lungs in DIC rats (Figure 5B), while oral treatment of um-PEA at 30 mg/kg ameliorated macroscopic alterations (Figure 5C).

In addition, H/E and PTAH staining showed a normal physiological structure of lungs taken from sham rats (Figure 5D,G and score in Figure 5J). In contrast, the parenchyma of the lungs following LPS infusion was significantly altered, with the presence of pulmonary microthrombus, as well as pulmonary edema and neutrophil infiltrations (Figure 5E,H and score in Figure 5J). Um-PEA treatment was able to reduce pulmonary histological alterations, including edema, inflammatory infiltrates, and microthrombus (Figure 5F,I and score in Figure 5J).

The presence of lung edema is also confirmed by the ratio of wet/dry (W/D) weight of the tissue, which showed a marked increase in the LPS group compared to sham animals, while um-PEA administration reduced the ratio of W/D weight of the tissue (Figure 5K).

### 2.5. Effect of um-PEA on MC Activation

**Figure 6:**
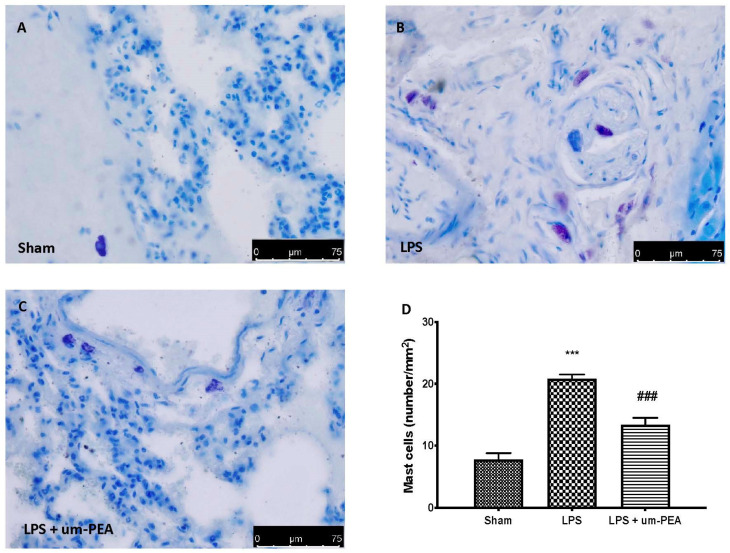
Toluidine blue staining: sham (**A**), LPS (**B**), LPS + um-PEA (**C**), mast cell count (**D**). A 40× magnification is shown (75-µm scale bar). *** *p* < 0.001 vs. sham; ### *p* < 0.001 vs. LPS.

Toluidine blue staining of lung sections was performed to evaluate MC degranulation. In the LPS group, we observed an increase in the number of MCs intact and degranulated (Figure 6B,D) compared to the control (Figure 6A,D). Um-PEA at the dose of 30 mg/kg significantly reduced MC activation (Figure 6C,D).

**Figure 7:**
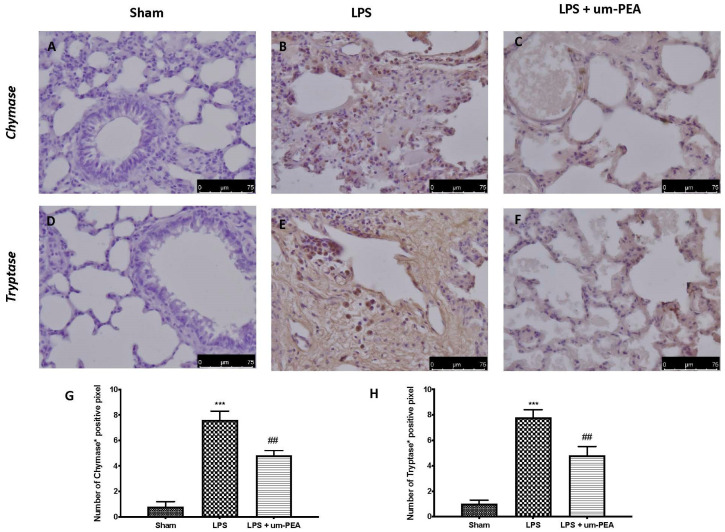
Immunohistochemical evaluation for chymase expression: sham (**A**), LPS (**B**), LPS + um-PEA (**C**); and tryptase expression: sham (**D**), LPS (**E**), LPS + um-PEA (**F**). Graphical quantification of chymase (**G**) and tryptase (**H**) expression in lung. A 40× magnification is shown (75-µm scale bar). *** *p* < 0.001 vs. sham; ## *p* < 0.01 vs. LPS.

To confirm the reduction in MC degranulation, we evaluated the expression of chymase and tryptase, two mast-cell-derived serine peptidases, in lungs. Immunohistochemical staining showed an important increase in chymase (Figure 7B,G) and tryptase (Figure 7E,H) expression in the LPS group compared to sham rats (Figure 7A,G,D,H, respectively); in contrast, um-PEA significantly reduced the expression of both markers (Figure 7C,G for chymase and Figure 7F,H for tryptase).

## 3. Discussion

DIC is a disease characterized by systemic activation of coagulation, potentially resulting in thrombotic occlusion of small- and medium-sized vessels [14]. At the same time, ongoing consumption of PLT and coagulation proteins causes thrombocytopenia and low levels of clotting factors, which may cause profuse hemorrhagic complications, thereby leading to organ dysfunction [22]. Experimental DIC in animals was induced by continuously infusing them with LPS for 4 h, which is an endotoxin present on the outer membrane of Gram-negative bacteria [8]. The presence of LPS in the bloodstream causes, beyond the DIC, fever, hypotension, cytokine production, MOF, and, in severe cases, septic shock and death [11]. The incidence of DIC is 30–50% in sepsis; in patients with sepsis and DIC, mortality is almost two times higher as compared with patients who do not have DIC [23]. In the pathophysiology of LPS-induced DIC, the so-called “cytokine storm” is also involved. Indeed, LPS induces monocytes and endothelial cells to release cytokines, such as TNFα, IL-1β, and IL-6, that, at high enough levels, have systemic effects [11]. This overproduction of proinflammatory cytokines causes coagulation activation and impairment of fibrinolysis in LPS-induced DIC, thus confirming cross-signaling between the pathways mediating coagulation and inflammation [24,25]. The imbalance between anticoagulant and procoagulant mechanisms in the blood and widespread systemic inflammation leads to a drop in blood pressure, inadequate removal of fibrin, and deposition of fibrin in the microvasculature [26,27]. Microvascular thrombosis contributes to the development of organ dysfunction, especially in the lung.

It is important to underline that, in the last two years, DIC has received particular attention as it is closely related to COVID-19 [3]. This experimental model of DIC has implications for understanding cardiovascular and pulmonary mortality in the COVID-19 pandemic. Indeed, activation of the coagulant pathway accompanied by excessive inflammatory/immune reactions (the so-called cytokine storm) and progression to MOF represent the principal causes of death [28,29]. In particular, the complications seen in patients with COVID-19 show respiratory failure due to marked microvascular thrombosis associated to extensive alveolar inflammation up to pulmonary tissue damage [30,31].

Therapeutically, supplementation with blood components, anticoagulants, and coagulation inhibitors has been used to treat DIC [32,33]. They aim to inhibit the activation of coagulation and prevent the formation of thrombi. However, bleeding problems often complicate the choice of treatment for DIC. Besides, based on pathophysiological concepts, coagulation inhibition combined with anti-inflammatory properties is regarded as an attractive therapeutic approach for human sepsis. Therefore, the type of medical intervention used to simultaneously alleviate the activation of inflammation and coagulation was considered an interesting prospect. We designed the present study to offer a different alternative to the traditionally used medications by exploiting well-known anti-inflammatory properties of um-PEA [19,34,35,36]. The objective of this study was to explore the relationship between the balance of fibrinolysis/coagulation, the cytokine storm, and lung dysfunction in the LPS-induced model of DIC, and to explore possible pathophysiological mechanisms. As previously said, proinflammatory cytokines play an important role in pathologic processes that contribute to the formation of microthrombus in DIC models [37]. Therefore, we speculated that reduced secretion of these proinflammatory factors would be advantageous for the prevention and treatment of DIC. Several studies have demonstrated that um-PEA could regulate the release of proinflammatory cytokines via several effector mechanisms on immune cells [18,38,39]. Hence, we investigated the effectiveness of um-PEA treatment in ameliorating LPS-induced DIC.

All physiological coagulation pathways are significantly compromised in DIC [40,41]. Markedly decreased plasma PLT counts were observed, as were significantly depressed plasma fibrinogen levels in LPS rats. The PT index was significantly increased after LPS infusion, indicating that the coagulation factor was greatly reduced and, consequently, the PT was prolonged. In the fibrinolysis phase induced by LPS, the body produced a large amount of fibrin degradation products, so the APTT was extended [42,43]. In contrast, all indexes were significantly improved, as shown by a decrease in upregulation of PT and APTT after um-PEA treatment, indicating that um-PEA has a good regulatory effect on the disorder of the coagulation and fibrinolytic system in DIC. Additionally, plasma levels of D-dimer are regarded as one of the most useful markers in the diagnosis of DIC. D-Dimer is a fragment of fibrin produced in the blood when fibrin crosslinked with factor XIII is degraded by plasmin [44]. That is, thrombus is created in the body when coagulation is activated, and it is decomposed when fibrinolytic activity is activated. Thus, elevated plasma D-dimer levels are considered to indicate the presence of DIC [45]. Consistent with this, our result showed that consumption of DD was decreased after treatment with um-PEA at the dose of 30 mg/kg.

Ongoing activation of platelets with coagulation factor consumption can contribute to the derangement of coagulation and fibrinolysis, leading to microvascular fibrin thrombi and proneness to severe inflammatory reaction [46]. Activation of the inflammatory cascade impacts the coagulation pathway, and vice versa. Widespread involvement of endothelium and monocytes/tissue macrophages, together with the more generalized activation of inflammation and coagulation, lead to the overproduction of inflammatory cytokines [5]. In our study, we observed high levels of inflammatory cytokines, such as IL-1β, IL-6, TNF-α, and IFN-γ, after intravenous infusion of LPS for 4 h, both in blood and lung tissue. Moreover, the production of inflammatory cytokines and mediators is linked with activation upstream of NF-κB, the principal inflammatory pathway associated with inflammation. A Western blot analysis confirmed that continuous infusion of LPS activated the NF-κB pathway. In contrast, treatment with um-PEA efficaciously reduced IκBα degradation and NF-κB nuclear translocation in lung sample, and, consequently, the overproduction of inflammatory cytokines, confirming the strong anti-inflammatory effect of um-PEA.

Furthermore, it had been reported that inflammation-related coagulopathy causes the formation and deposition of fibrin, leading to microvascular thrombosis in different organs, especially in the lungs [3,47]. We hypothesized that mitigation of inflammation can attenuate lung damage and dysfunction; so, we wanted to test the well-known anti-inflammatory properties of um-PEA in rats subjected to DIC. Lung tissues were stained by H/E, as well as phosphotungstic acid hematoxylin (PTAH), to evaluate the severity of inflammation and organ failure. Our results showed fibrin-positive staining and bleeding in pulmonary alveoli in rats following LPS infusion, while treatment with um-PEA decreased marked fibrin deposition, as well as the macroscopic alterations.

In the pathophysiology of the multisystemic reaction, MCs play a key role; in fact, their activation leads to the consequent degranulation and release of inflammatory mediators, including proteases and proinflammatory cytokines. The roles of MCs in coronavirus-induced inflammation and cytokine storm have been recently discussed [48,49]. Hyperactive MCs can get into a continuous activation loop, resulting in cytokine storms, the fluid build-up and pulmonary damage often seen in severe COVID-19 patients [50]. Additionally, MCs also express many serine proteases, which are necessary for SARS-CoV-2 infection [51]. Among these, chymase and tryptase, proteases exclusively of MC origin, are also involved in the activation of the coagulation system. In particular, MC chymase is responsible for the degradation of both fibrinogen and fibrin, while MC tryptase has been shown to participate in fibrinolysis through plasmin activation and by facilitating the degradation of fibrinogen [51,52]. According to the literature, our results showed a significant increase in both the number of MCs and the release of proteases, as demonstrated by blue toluidine staining and immunohistochemistry analysis for chymase and tryptase. On the other hand, the main mechanism of action of um-PEA, belonging to the ALIAmides family, relies on the downmodulation of MC degranulation. Hence, as can be expected, um-PEA treatment was able to reduce the number of intact and degranulated MCs. These results are also confirmed by the reduction in the expression of chymase and tryptase secreted by activated MCs.

## 4. Materials and Methods

### 4.1. Animals

Sprague–Dawley rats (200–250 g, male; Envigo, Italy) were housed in a controlled environment with free access to typical rodent diet and water. This study was approved by the University of Messina Review Board for the care of animals (approval number 499/2018-PR). Animal care conformed to Italian and European regulations on the use of animals for experimental and scientific purposes (D.Lgs 2014/26 and EU Directive 2010/63).

### 4.2. Induction of DIC

DIC model was induced in rats by continuously infusing them with LPS (30 mg/kg, dissolved in 10 mL sterile saline; Escherichia coli 055: B5; Sigma-Aldrich, St. Louis, MO, USA) via the tail vein for up to 4 h [2,53]. The control group (sham) was injected with the same volume of saline.

### 4.3. Experimental Groups

Rats were randomly distributed into several groups (n = 15 for each):

- LPS group: rats received infusion of LPS, as previously described;
- LPS + um-PEA group: rats received infusion of LPS, as previously described, and were treated with um-PEA (30 mg/kg) by oral gavage 30 min before and 1 h after the start of intravenous infusion of LPS;
- Sham group: identical to the LPS group, but animals received saline (0.9% *w*/*v*) instead of LPS;
- Sham + um-PEA group: identical to the sham group, but rats were treated with um-PEA (30 mg/Kg) by oral gavage 30 min before and 1 h after the start of experiment.

Since no significant changes were found between the sham groups for coagulation markers and cytokine levels, we present data of the sham + saline group.

The dose of um-PEA was selected based on previous studies [54,55]. Rats were euthanized 12 h after LPS infusion and lungs were harvested for further analysis.

### 4.4. Blood Sampling and Parameter Measurement

Blood was withdrawn from the abdominal aorta of rats under anesthesia into the 4% sodium citrated tubes after LPS or saline infusion at 0 h (before), 4 h, 8 h, and 12 h, and analyzed within 2 h from blood collection. Platelet (PLT) counts were performed with an automated device for animals (Celltac, MEK-5128, Nihon Kohden Co., Tokyo, Japan) [37]. Fibrinogen (Fib) levels, prothrombin time (PT), and activated partial thromboplastin time (APTT) were measured using standard kits for a Clot 2 coagulometer (SEAC diagnostics, Florence, Italy) [56]. D-dimer levels were determined by the quantitative latex agglutination test (Diatron, Tokyo, Japan) [37].

### 4.5. Cytokine Levels

Plasma and lung levels of TNF-α, IL-1β, IL-6, and IFN-γ, were measured by the ELISA kits (R&D Systems, Minneapolis, MN, USA) [37,57,58]. All protocols were performed by following the manufacturer’s instructions.

### 4.6. Western Blot Analysis

Western blot analysis was performed as previously described [59]. The following primary antibodies were used: anti-IκBα (1:500, Santa Cruz Biotechnology (SCB), #sc1643), anti-NF-κB p65 (1:500, SCB, #sc8008), anti-β-actin (1:5000; SCB, #sc8432), and anti-lamin A/C antibody (1:5000; Sigma-Aldrich, St. Louis, MO, USA). Protein expression was quantified by densitometry with BIORAD ChemiDocTM XRS + software and normalized to housekeeping genes β-actin and lamin A/C as previously reported [60].

### 4.7. Histopathology

Lung tissue sections (7 μm) were stained with hematoxylin/eosin (H/E) and phosphotungstic acid hematoxylin (PTAH) for histopathological examination. Sections were examined using a Leica DM6 microscope (Leica Microsystems SpA, Milan, Italy) associated with Leica LAS X Navigator software (Leica Microsystems SpA, Milan, Italy) [61]. Every piece was viewed at a magnification of 20× for H/E and 40× for PTAH, and morphological changes were evaluated by two blinded investigators. The grading scale to score histopathologic findings was determined as previously described [62].

### 4.8. Measurement of Lung Edema

At the end of experiment, wet lung weights were recorded. The lungs were subsequently dried for 48 h at 80 °C and weighed again. The water content of the tissue was calculated as the ratio of wet/dry weight of the lung [62].

### 4.9. Staining of Mast Cells (MCs)

At the end of experiment, for identification of MCs, lung tissue sections (7 μm) were stained with toluidine blue, as described previously [63]. Every section was observed at a magnification of 100×, using a Leica DM6 microscope (Leica Microsystems SpA, Milan, Italy) associated with Leica LAS X Navigator software (Leica Microsystems SpA, Milan, Italy).

### 4.10. Immunohistochemical Analysis

Immunohistochemical analysis was performed as previously described. Lung sections were incubated overnight with primary antibodies: anti-MC chymase (1:100, SCB, #sc59586) and anti-MC tryptase (1:100, SCB #sc59587) antibodies. Images were collected using a Leica DM6 microscope (Leica Microsystems SpA, Milan, Italy) following a typical procedure [64]. The histogram profile is related to the positive pixel intensity value obtained [65].

### 4.11. Statistical Evaluation

All values are expressed as mean ± standard error of the mean (SEM) of N observations. The images shown are representative of the least 3 experiments performed on diverse experimental days on tissue sections collected from all animals in each group. For in vivo studies, N represents the number of animals used. The results were analyzed by one-way ANOVA, followed by a Bonferroni post hoc test for multiple comparisons. A *p*-value less than 0.05 was considered significant.

## 5. Conclusions

In conclusion, this study demonstrates that um-PEA has the function of alleviating the complications associated to LPS-induced DIC, such as coagulation disturbances, cytokine storm, or intravascular formation of fibrin deposition, at least in the lung. Furthermore, um-PEA downregulates MC degranulation and its mediators, involved in the activation of the coagulation system, in lung tissues. Further studies will be conducted to better investigate the mechanism of action of um-PEA in experimental models of DIC. Importantly, PEA lacks acute and chronic toxicity, and it is well tolerated and no interaction with other ongoing therapy was reported. Therefore, we propose that um-PEA can be considered as a potential therapeutic approach in the treatment of CID, and, therefore, may have clinical implications in conditions associated to coagulopathy and lung dysfunction, such as COVID-19.

## Data Availability

The datasets used in the current study are available from the corresponding author on reasonable request.
